# Bacterial Load of Pneumococcal Serotypes Correlates with Their Prevalence and Multiple Serotypes Is Associated with Acute Respiratory Infections among Children Less Than 5 Years of Age

**DOI:** 10.1371/journal.pone.0110777

**Published:** 2014-10-31

**Authors:** Bhim Gopal Dhoubhadel, Michio Yasunami, Hien Anh Thi Nguyen, Motoi Suzuki, Thu Huong Vu, Ai Thi Thuy Nguyen, Duc Anh Dang, Lay-Myint Yoshida, Koya Ariyoshi

**Affiliations:** 1 Department of Clinical Medicine, Institute of Tropical Medicine, Nagasaki University, Nagasaki, Japan; 2 Department of Bacteriology, National Institute of Hygiene and Epidemiology, Hanoi, Vietnam; 3 Department of Microbiology, Khanh Hoa General Hospital, NhaTrang, Vietnam; 4 Department of Pediatric Infectious Diseases, Institute of Tropical Medicine, Nagasaki University, Nagasaki, Japan; 5 Graduate School of Biomedical Sciences, Nagasaki University, Nagasaki, Japan; University Hospital San Giovanni Battista di Torino, Italy

## Abstract

**Background:**

Among pneumococcal serotypes, some serotypes are more prevalent in the nasopharynx than others; determining factors for higher prevalence remain to be fully explored. As non-vaccine serotypes have emerged after the introduction of 7-valent conjugate vaccines, study of serotype specific epidemiology is in need. When two or more serotypes co-colonize, they evolve rapidly to defend host's immune responses; however, a clear association of co-colonization with a clinical outcome is lacking.

**Methods:**

Children less than 5 years old who were admitted to hospital due to acute respiratory infections (ARI) (n = 595) and healthy children (n = 350) were recruited. Carriage of pneumococcus was determined by culture and lytA PCR in the nasopharyngeal samples. Serotype/serogroup detection and its quantification were done by the nanofluidic real time PCR system. Spearman's correlation and logistic regression were used to examine a correlation of serotype/serogroup specific bacterial load with its prevalence and an association of co-colonization with ARI respectively.

**Results:**

Serotype/serogroup specific bacterial load was correlated with its prevalence, both in ARI cases (Spearman's rho = 0.44, n = 186; P<0.0001) and healthy children (Spearman's rho = 0.41, n = 115; P<0.0001). The prevalence of multiple serotypes was more common in ARI cases than in healthy children (18.5% vs 7.1%; aOR 2.92, 95% CI: 1.27–6.71; P = 0.01). The dominant serotype in the co-colonization had a 2 log10 higher bacterial load than the subdominant serotype, both in ARI cases (P<0.001) and healthy children (P<0.05).

**Conclusions:**

High bacterial load in the nasopharynx may help transmit pneumococci among hosts, and increase the chance of successful acquisition and colonization. Co-colonization of multiple serotypes of pneumococci is linked with ARI, which infers the interactions of multiple serotypes may increase their pathogenicity; however, they may compete for growth in number.

## Introduction

*Streptococcus pneumoniae* (pneumococcus) is a major cause of life threatening diseases including pneumonia, sepsis and meningitis in children worldwide [1]. Pneumococcus has distinct polysaccharide capsule which characterizes its more than 90 serotypes. It colonizes in the nasopharynx, which is the precursor for pneumococcal diseases and the source for transmission among people [2]. The emergence of non-vaccine serotypes (19A, 35B) after the introduction of 7-valent pneumococcal conjugate vaccine (PCV7) has become a concern for future epidemiology of pneumococcal diseases, and it highlights the need of serotype specific study of this common pathogen [3], [4].

The prevalence of a serotype in the nasopharynx is inversely correlated with the invasiveness of the serotype [5]. Less invasive serotypes such as 6A, 6B, 19F, 23F tend to colonize more frequently and maintain the carriage for longer time; while the more invasive serotypes such as 1, 4, 5, 7F tend to colonize less frequently and maintain colonization for less duration [6], [7]. The pneumococcal capsule, which is a major virulent factor, determines the serotype. It is found the thickness of the capsule positively correlates with the prevalence of the serotype/serogroup [8]. Furthermore, the capacity to grow of a serotype is correlated with its prevalence in an in vitro study [9]; however, it is unknown whether similar relationship exists in the natural niche in humans.

Co-colonization of multiple pneumococcal serotypes in the nasopharynx affects vaccine serotype replacement, carriage detection and pneumonia diagnosis [10]. Accurate determination of co-colonization and its role in pathogenesis are difficult to establish by using conventional serotyping method because of its low sensitivity and tedious laboratory work; therefore, a highly sensitive and specific molecular method is in need to detect multiple serotypes accurately and efficiently [11], [12]. Although varied prevalence rates of co-colonization have been reported from different parts of the world [11]–[15], an epidemiological evidence of their association with a clinical outcome is lacking. Some epidemiological models have demonstrated that the serotypes compete among themselves for acquisition and persistence of colonization in the nasopharynx [16], [17]; however, it is yet to be demonstrated quantitatively in humans.

In this study, we applied a highly sensitive and specific novel nanofluidic real time PCR system to identify serotypes/serogroups and quantify their bacterial load in nasopharyngeal samples of hospitalized acute respiratory infections (ARI) cases and healthy children. We aimed to assess the correlation of bacterial load of specific serotypes/serogroups with the prevalence and compare the prevalence of co-colonization of multiple serotypes between these two groups of children.

## Methods

### Study design

Acute respiratory infections (ARI) cases, as defined by World Health Organization (WHO), were recruited in the Department of Pediatrics, Khanh Hoa General Hospital, Nha Trang City, Vietnam from 07/04/2008 to 31/03/2009. The hospital has 750 beds, and is the only hospital that provides inpatient care for sick children in Nha Trang City. The participants were children under 5 years old from the study area, who were admitted to the pediatric ward during the study period with acute respiratory infections (ARI) defined by cough and/or difficulty in breathing (WHO). We also included healthy children to compare bacterial load and serotype distribution with ARI cases. Healthy children, who did not have fever, signs of respiratory infections or history of antibiotic intake in the month preceding the day of enrollment, were recruited from two communes in the study area. They were selected randomly during January 2008 from the under 5 years old children using the census data. Informed written consent was taken from the parents of the hospital admitted ARI cases and the healthy children. Details of the study site were described elsewhere [18].

### Sample collection, storage and DNA extraction

A nasopharyngeal sample (NPS), (about 100 microliter in volume) was collected flexible dacron-tipped aluminum-shafted swabs (Copan, Brescia, Italy) from each participant according to the WHO protocol [19]. The samples were taken from ARI cases at the time of admission before they were treated with antibiotics. The samples were divided into two aliquots. One aliquot was sub-cultured onto 5% sheep blood agar and incubated overnight with 5% CO_2_ at 37°C. Alpha hemolytic colonies with morphology suggestive of *S. pneumoniae* and positive Optochin test were considered potential *S. pneumoniae* isolates. The other aliquot was stored at −80°C. At a later stage DNA was extracted, following the protocol of QIAGEN kit for the Gram-positive bacteria, directly from the nasopharyngeal samples of the children from whom potential *S. pneumoniae* had been cultured. DNA was stored at -80°C, except for transportation to Nagasaki on dry ice, till used for the lytA PCR and the nanofluidic real time PCR system.

### Identification of pneumococcus, its serotypes, quantification of bacterial load and definition of co-colonization of multiple serotypes

The DNA extract of the NPS from children with a potential *S. pneumoniae* culture positive isolate was analyzed by a lytA PCR that targeted the autolysin gene using the Light cycler II (Roche). Carriage of pneumococcus is defined as having positive lytA PCR NPS. The lytA positive samples were subjected to the nanofluidic real time PCR system for molecular serotyping. The nanofluidic real time PCR system can identify 50 serotypes in 29 groups, and it can detect minor population of multiple serotypes in co-colonization of pneumococci with the minimum level of detection of 30 to 300 copies. Total and specific serotype/serogroup bacterial loads were quantified using the standard curves. Details of the nanofluidic real time PCR system has been described elsewhere [20]. Samples positive for lytA and negative for all serotype/serogroup primer-pairs were grouped as non-typeable (NT). Co-colonization with multiple serotypes was defined as the presence of two or more serotypes/serogroups in a sample. In co-colonization the first serotype and second serotype were defined according to their bacterial loads; a serotype with the highest load was assigned as first serotype, with the second highest load as second serotype.

### Statistical analysis

The data of pneumococcal loads, DNA copies, were changed into log10 scale. To compare the groups, Wilcoxon rank sum test was used for the continuous variables and Chi-square or Fisher exact test was used for categorical variables. Logistic regression was used to test the effects of pneumococcal load and co-colonization as risk factors for hospitalized children due to ARI. Odds ratios were adjusted for age, sex and daycare attendance. To test the correlation of bacterial load and prevalence of individual serotypes, Spearman's correlation was used. Analyses were performed using Stata v12.1 (StataCorp, College Station, Taxes, USA). The database for the analysis was submitted as (Table S1, S2).

### Ethical approval

This study was approved by all the concerned research review boards: Nagasaki University Institutional Review Board Nagasaki, Japan; the National Institute of Hygiene and Epidemiology Scientific Review Committee, Hanoi and the Khanh Hoa Provincial Health Service Ethical Review Board, Nha Trang, Vietnam.

## Results

### Basic characteristics of ARI cases and healthy children

Among the hospital admitted ARI cases, 88.6% (527/595) were children less than 2 years old, proportion of male was 62% (369/595), and the median age was 10 months. Almost half, 45.5% of ARI cases had history of antibiotic use before admission. The proportion of chest X-ray confirmed pneumonia among them was 22.8% (136/595). Among healthy children, 55.4% (194/350) were under 2 years of old, proportion of male was 52.6% (184/350), and the median age was 19 months.

### Pneumococcal colonization and serotype distribution

**Figure 1:**
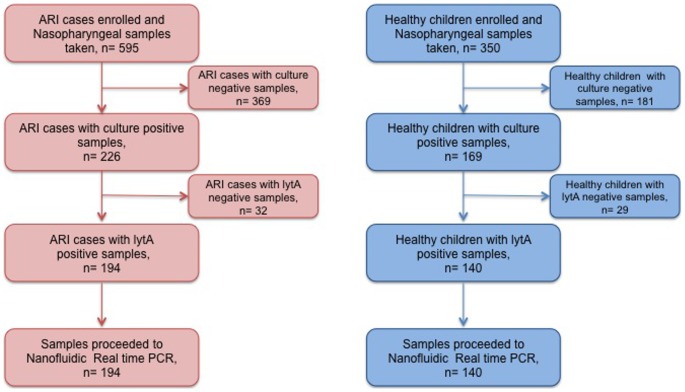
Participants recruited into the study, and their nasopharyngeal samples. Half of the aliquot of nasopharyngeal samples were cultured overnight in 5% sheep blood agar with 5% CO_2_ at 37°C. DNA was extracted directly from the other half of the aliquot of the nasopharyngeal samples which yielded alpha hemolytic colonies and positive Optochin test. DNA was subjected to lytA PCR to confirm pneumococcus; and lytA positive samples were then processed for molecular serotyping in the nanofluidic real time PCR system.

**Figure 2:**
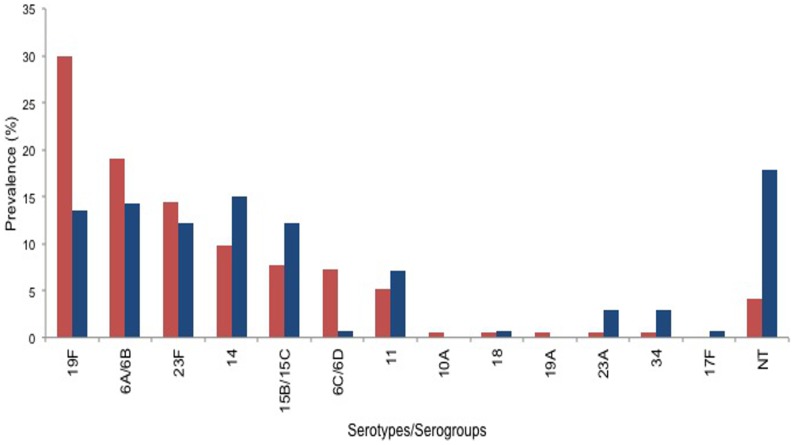
Distribution of serotypes/serogroups of pneumococcus in ARI cases and healthy children. Thirteen different serotypes/serogroups were detected; DNA samples which were positive for lytA (pneumococcus positive), but negative for tested 29 serotypes/serogroups were assigned as non-typeable (NT). Prevalence of each serotype/serogroup was calculated as proportion of total number of a serotype/serogroup to the total number of the lytA positive samples. Serotype/serogroup of ARI cases and healthy children were plotted in red and blue respectively. Proportion of serotype/serogroup covered by 13-valent conjugated vaccine (PCV13) was 74% in ARI cases and 55% in healthy children.

Pneumococcal carriage rate was 32.6% (194/595) in ARI cases and 40% (140/350) in healthy children (figure 1). The proportion of typeable serotypes in ARI cases and healthy children were 95.9% (186/194) and 82.1% (115/140) respectively. Thirteen serotypes/serogroups of pneumococcus were detected in ARI cases and healthy children (figure 2). Serotypes/serogroups 19F, 6A/6B, 23F and 6C/6D were more prevalent in hospitalized ARI cases while serotypes/serogroups 14, 6A/6B, 19F, 15B/15C, 11 and non-typeables (NT) were common in healthy children. The proportion of serotypes/serogroups covered by pneumococcal conjugate vaccines: 7-valent (PCV7), 10-valent (PCV10) and 13-valent (PCV13) were approximately equal to one another, which was about 74% in ARI cases and 55% in healthy children (this was an approximate estimation as a serogroup was treated as a serotype as required).

### Pneumococcal bacterial load

**Figure 3:**
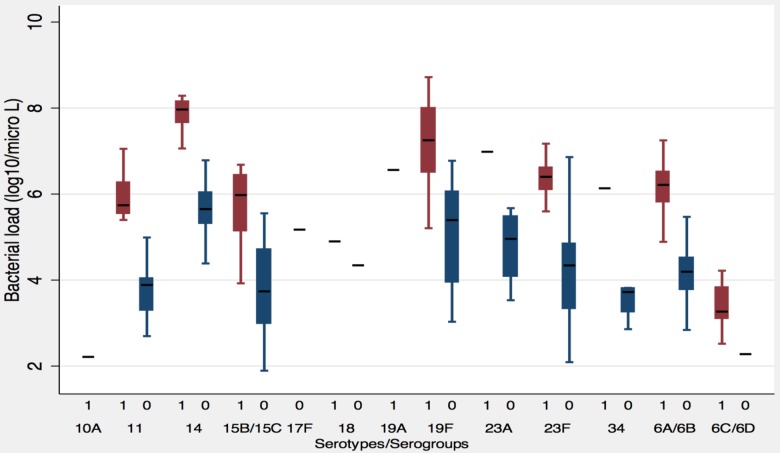
Bacterial load of specific serotypes/serogroups of pneumococcus in ARI cases and healthy children. Bacterial load of ARI cases (denoted by “1” in each serotype/serogroup) in red and healthy children (denoted by “0” in each serotype/serogroup) in blue, showed that the common serotypes 19F, 14, 23F, 6A/6B, 15B/15C had high bacterial load.

Higher bacterial load was associated with hospitalization due to ARI. The median bacterial load (total) was 6.61 log10/ml in ARI cases and 4.36 log10/ml in healthy children (OR = 9.96, 95%CI: 6.39–15.52; P<0.0001 and aOR = 9.07, 95%CI: 5.69–14.4; P<0.0001). There was no difference in median bacterial load between males and females both in ARI cases (6.62 log10 in male Vs. 6.59 log10 in female; p-value: 0.76) and healthy children (4.36 log10 in male Vs. 4.32 log10 in female; p-value: 0.86). Bacterial loads of specific serotypes/serogroups were higher in ARI cases than healthy children in all detected serotypes (Figure 3). Serotypes 14, 19F, 23F, 6A/6B had higher median bacterial load than other serotypes in ARI cases while serotypes 14, 19F, 23A, 23F had higher load in healthy children. The bacterial load of serotype 6C/6D was found to be lower than other common serotypes in ARI cases.

Serotype/serogroup specific bacterial load was significantly higher in vaccine serotypes. When we compared the bacterial load of vaccine specific serotypes and non-vaccine serotypes, the load was higher in vaccine serotypes both in ARI cases: 6.61 log10 in vaccine serotypes and 5.51 log10 in non-vaccine serotypes (P<0.0001), and in healthy children: 4.68 log10 in vaccine serotypes and 3.81 log10 in non-vaccine serotypes (P = 0.0001).

### Correlation between bacterial load of serotypes and their prevalence

**Figure 4:**
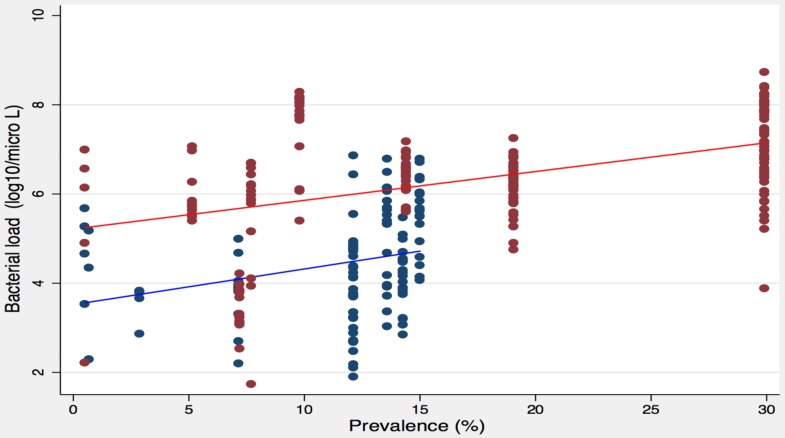
Relationship between bacterial load of specific serotypes/serogroups and their prevalence. Bacterial load of each of specific serotypes/serogroups was plotted against its prevalence. Both in ARI cases (red) and in healthy children (blue), a positive correlation was found. Each dot represents an ARI case or a healthy child in the plot, and the red and blue lines are the fitted values for ARI cases and healthy children respectively. Spearman's rho was 0.44 (n = 186; P<0.0001) and 0.41 (n = 115; P<0.0001) for ARI cases and healthy children respectively.

Serotype/serogroup specific bacterial load was positively correlated with serotype/serogroup prevalence. Serotype/serogroup specific bacterial load of individual NPS samples from ARI cases and healthy children were plotted against the prevalence of individual serotype/serogroup (i.e., proportion of the serotypes/serogroups in carriage positive samples). We found that the serotype/serogroup specific bacterial load had positive correlation with its prevalence, both in ARI cases (Spearman's rho = 0.44, n = 186, P<0.0001) and healthy children (Spearman's rho = 0.41, n = 115, P<0.0001) (Figure 4).

### Co-colonization with multiple serotypes

**Figure 5:**
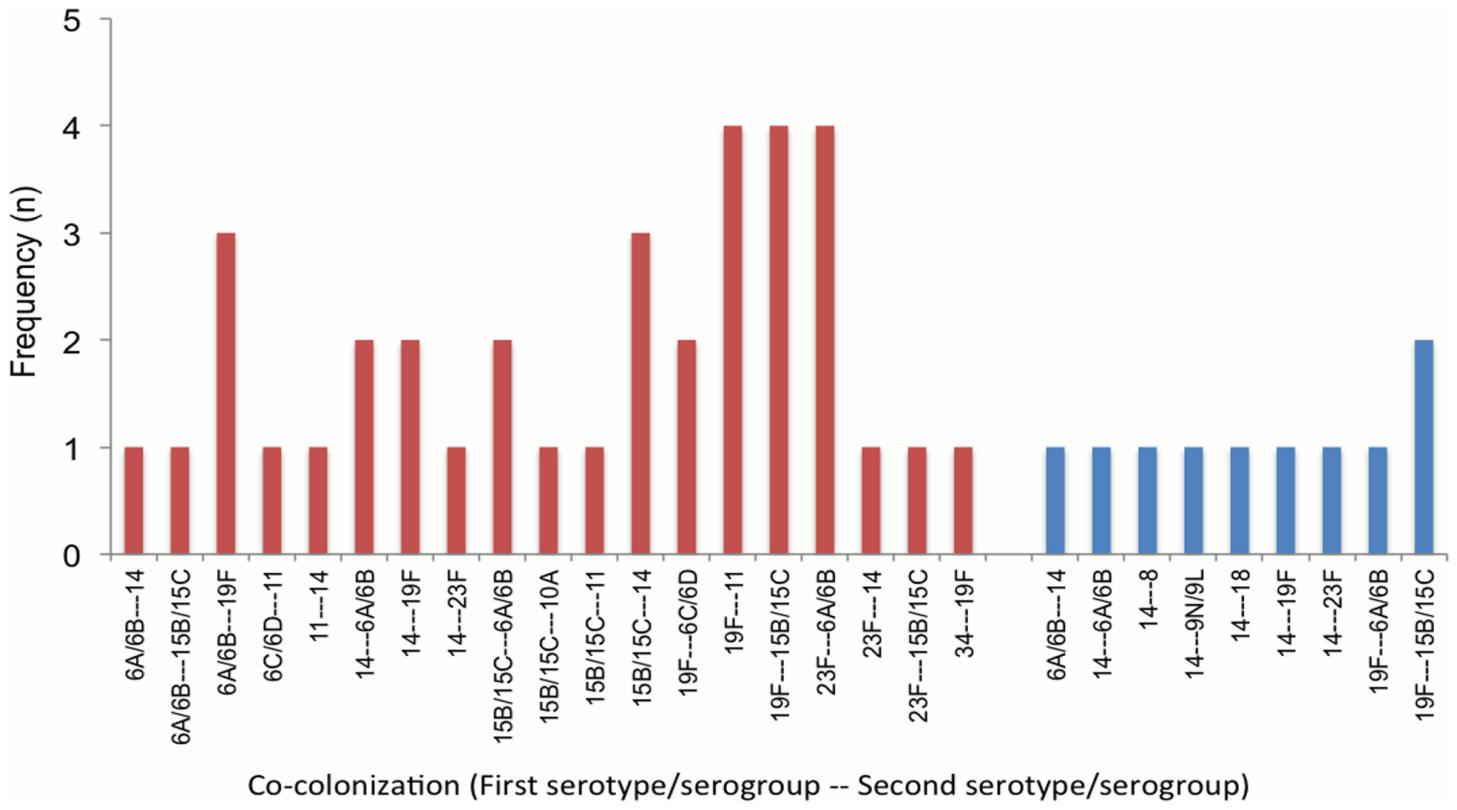
Distribution of co-colonization of multiple serotypes of pneumococcus. In ARI cases (red) co-colonization was detected in 36 samples out of 194 lytA positive samples (18.5%), while in healthy children (blue) it was detected in 10 samples out of 140 (7.1%) lytA positive samples. The odds ratio, adjusted for age, sex and daycare, was 2.92 (95%CI 1.27–6.71; P = 0.012). The serotypes/serogroups were positioned first and second according to their bacterial load.

Co-colonization of multiple serotypes of pneumococci was associated with ARI. Co-colonization of multiple-serotypes was detected in 18.5% (n = 36/194) in ARI cases and 7.1% (n = 10/140) in healthy children (OR 2.96, 95%CI 1.41–6.19; P = 0.004). When adjusted for age, sex and daycare, the adjusted odds ratio (aOR) was 2.92 (95%CI 1.27–6.71; P = 0.012). Co-colonization of serotypes 19F and 11, 19F and 15B/15C, 23F and 6A/6B occurred more frequently in ARI cases. We observed none of the serotypes involved in co-colonization were 19A or 35B (Figure 5). Co-colonization of pneumococcus occurred in 18.56% (18/97) of ARI cases when pre-hospital antibiotics had been used and 38.71% (12/31) when no antibiotics were used (P = 0.002) [Unknown antibiotic status in 9.09% (6/66) of ARI cases]. The prevalence of co-colonization was found to be higher 32.5% (13/40) in age group of less than 6 months of age than 14.94% (23/154) in age group of 6 or more than 6 months of age in ARI cases (P = 0.01). But, we did not find any such difference in healthy children among these age groups, 0% (0/16) versus 7.04% (10/124) (P = 0.60).

**Figure 6:**
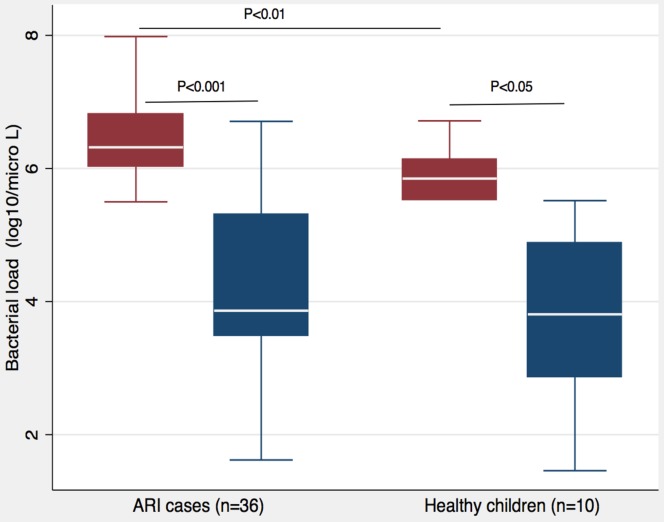
Dominance of one serotype/serogroup over the other in co-colonization of multiple serotypes/serogroups. Among two serotypes/serogroups present in a co-colonization, one serotype/serogroup (red) was found to be dominant by having 100 folds (2 log10) higher bacterial load than the other subdominant (blue) serotype/serogroup both in ARI cases and healthy children.

One serotype/serogroup dominated the other serotype/serogroups in co-colonization. In co-colonization, the serotype/serogroup specific bacterial load was 2.45 log10 higher in the dominant serotypes than the subdominant serotypes in ARI cases, while it was 2.04 log10 higher in healthy children (Figure 6). The dominant serotype was a vaccine serotype in 100% (10/10) of co-colonization in healthy children (P = 0.003); while it was vaccine serotypes only in 72.22% (26/36) of co-colonization in ARI cases (P = 0.76). The median total bacterial load was higher, 4.81 log10 versus 4.31 log10 (P = 0.03), when co-colonization of multiple serotypes was present as compared to single serotype colonization in healthy children; however, no significant difference was found among them in ARI cases (6.65 log10 versus 6.58 log10, P = 0.59).

## Discussion

Our data suggest two major findings. First, a positive correlation of serotype/serogroup specific bacterial load with prevalence of serotypes/serogroups, which may help to understand why some serotypes of pneumococcus are successful for colonization and maintenance of the carriage for longer time. The second finding, an association of co-colonization of multiple serotypes with acute respiratory infections in children may infer a role of multiple serotypes of pneumococci in pathogenesis of ARI in children. Both of these findings are novel in pneumococcal pathogenesis and epidemiology in humans.

It is known that the prevalence of serotypes is inversely correlated with their serotype specific invasiveness [5]. It is found that some serotypes/serogroups: 1, 4, 5, 7F are more invasive but less prevalent in the nasopharynx than other serotypes/serogroups: 6A, 6B, 19F, 23F, which are more prevalent but less invasive [5]. Contributing factors for this inverse relationship of prevalence and invasiveness remains to be fully explained. It is found that colonizing serotypes: 19F, 6A, 6B, 23F have higher serotype specific rates of acquisition of colonization and longer duration of carriage than invasive serotypes: 1, 4, 5, 7F in children [6], [7]. This shows that the colonizing serotypes are more capable to colonize and maintain the carriage than invasive serotypes. In this regard, our finding of positive correlation of serotype/serogroup specific bacterial load with prevalence of serotype/serogroup may explain that the higher numbers of bacteria in the nasopharynx may help them to transmit from one host to other, so that they have higher chance of colonization, and their ability to grow in number may help to maintain the carriage for longer time against the normal mucosal clearance of the host.

Some in vitro studies show that serotype specific growth of pneumococcus is positively correlated with prevalence of the serotype [8], [9]. Hathaway et al found that high carriage prevalence serotypes (6B, 9V, 19F, 23F) can produce their capsules that are less metabolically demanding, and they can grow even in nutritionally poor environment [9]. These in vitro findings match with our in vivo findings of high bacterial load of common serotypes/serogroups and their correlation with prevalence. Less prevalent but more invasive serotypes are characterized by poor growth, longer lag phase in bacterial growth [21], thinner capsule size and more prone to be killed by neutrophils than high prevalence serotypes [8].

We found the occurrence of co-colonization with multiple serotypes of pneumococcus was twice as common in hospitalized ARI cases as in healthy children. This is the first time to our knowledge that such an association of co-colonization of multiple serotypes of pneumococci with ARI is reported. Although prevalence of co-colonization has been reported to occur from 1.3% to 39% in children by using different methods in various settings [11]–[15], none has reported an association with a clinical outcome. Non-vaccine serotypes such as 19A and 35B, which have emerged after the introduction of PCV7 vaccine [3], [4], were not detected in co-colonization both in ARI cases and healthy children in our study. Although the number of children with co-colonization was relatively small, our data discourage the “unmasking phenomena” for emergence of non-vaccine serotypes [22]. The potential role of co-colonization in the emergence of 19A due to serotype replacement is partly explained by capsule switching phenomenon at the capsular locus by recombination [23], [24]. As pneumococcus is highly recombinogenic and transformable bacteria, probability is high for genetic exchange when two or more pneumococcal serotypes inhabit at the same time in the nasopharynx [10]. This is true for evolution of not only vaccine-escape serotypes but also for the antibiotic resistant serotypes [24]. When multiple serotypes co-colonize, the genetic reservoir expands so called “Supragenome” will advance the microevolution of the pathogen and allow continued survival by evasion of serotype-specific immune response and adaptation by genetic change, which is demonstrated by the co-colonization of penicillin sensitive and penicillin resistance pneumococci at the same time [25]. Besides, it is found that in mouse model, pneumococci form a biofilm in the nasopharynx when multiple strains are present. The transformation efficiency becomes very high in the biofilm with multiple serotypes, so that the antibiotic resistance can easily be spread among the serotypes. Hence, co-colonization of multiple serotypes confers the development of supra-virulence and fitness in the pathogen for survival in the harsh environment of host [26].

We have further demonstrated the dominance of one serotype over the other in bacterial load when co-colonization is present. This difference in pneumococcal load among serotypes/serogroups in a co-colonization may suggest that there may be a competition among the serotypes for their growth due to limited nutrients and space. In mouse model, intra-species competition among pneumococci is demonstrated and found mediated by bacteriocin [27], [28]. Epidemiological models also suggest the existence of competition among serotypes for initiation and persistence of colonization in children [16], [17]. A study with mathematical modeling shows that direct (physical) competition and indirect (antibody mediated) competition do exit among the serotypes of pneumococcus in co-colonization [29]. We considered the 100 fold higher bacterial load, which we found in the dominant serotype, is due to direct competition as naturally acquired immune response due to colonization lasts for a short duration in unvaccinated young children. This is consistent with the finding of the mathematical modeling [29].

This study has limitations. Due to logistic problems, we were unable to bring all nasopharyngeal samples to Nagasaki from Vietnam for DNA extraction and nanofluidic real time PCR. First, we screened all the samples by culture, and only samples that grew alpha hemolytic colonies and Optochin sensitive isolates were brought to Nagasaki for molecular assays. It may have decreased the overall sensitivity of detection of pneumococcus and carriage rate. Minimum level of detection of the nanofluidic real time PCR was 30-300 copies per reaction; other limitations of the nanofluidic real time PCR have been described elsewhere [20].

## Conclusions

This study shows a positive correlation of serotype/serogroup specific bacterial load with serotype/serogroup prevalence of pneumococcus in children. Higher bacterial load of a serotype in the nasopharynx may be an attributing factor for higher transmission of the serotype. The association of multiple serotypes of pneumococcus with ARI shows its link with increased pathogenicity, and dominance of one serotype over the other may infer the competition among serotypes when multiple serotypes are present in the nasopharynx.

## Supporting Information

Table S1 **Database of ARI and healthy children.**
(XLSX)

Table S2 **Data dictionary file of database of ARI and healthy children.**
(DOCX)

## References

[1] O'Brien KL, Wolfson LJ, Watt JP, Henkle E, Deloria-Knoll M, et al (2009) Burden of disease caused by Streptococcus pneumoniae in children younger than 5 years: global estimates. Lancet 374: 893–902. 19748398 10.1016/S0140-6736(09)61204-6

[2] Simell B, Auranen K, Kayhty H, Goldblatt D, Dagan R, et al (2012) The fundamental link between pneumococcal carriage and disease. Expert Rev Vaccines 11: 841–855. 22913260 10.1586/erv.12.53

[3] Weinberger DM, Malley R, Lipsitch M (2011) Serotype replacement in disease after pneumococcal vaccination. Lancet 378: 1962–1973. 21492929 10.1016/S0140-6736(10)62225-8 PMC3256741

[4] Huang SS, Hinrichsen VL, Stevenson AE, Rifas-Shiman SL, Kleinman K, et al (2009) Continued impact of pneumococcal conjugate vaccine on carriage in young children. Pediatrics 124: e1–11. 19564254 10.1542/peds.2008-3099 PMC2782668

[5] Brueggemann AB, Peto TE, Crook DW, Butler JC, Kristinsson KG, et al (2004) Temporal and geographic stability of the serogroup-specific invasive disease potential of Streptococcus pneumoniae in children. J Infect Dis 190: 1203–1211. 15346329 10.1086/423820

[6] Abdullahi O, Karani A, Tigoi CC, Mugo D, Kungu S, et al (2012) Rates of acquisition and clearance of pneumococcal serotypes in the nasopharynges of children in Kilifi District, Kenya. J Infect Dis 206: 1020–1029. 22829650 10.1093/infdis/jis447 PMC3433858

[7] Tigoi CC, Gatakaa H, Karani A, Mugo D, Kungu S, et al (2012) Rates of acquisition of pneumococcal colonization and transmission probabilities, by serotype, among newborn infants in Kilifi District, Kenya. Clin Infect Dis 55: 180–188. 22523268 10.1093/cid/cis371 PMC3381638

[8] Weinberger DM, Trzcinski K, Lu YJ, Bogaert D, Brandes A, et al (2009) Pneumococcal capsular polysaccharide structure predicts serotype prevalence. PLoS Pathog 5: e1000476. 19521509 10.1371/journal.ppat.1000476 PMC2689349

[9] Hathaway LJ, Brugger SD, Morand B, Bangert M, Rotzetter JU, et al (2012) Capsule type of Streptococcus pneumoniae determines growth phenotype. PLoS Pathog 8: e1002574. 22412375 10.1371/journal.ppat.1002574 PMC3297593

[10] Shak JR, Vidal JE, Klugman KP (2013) Influence of bacterial interactions on pneumococcal colonization of the nasopharynx. Trends Microbiol 21: 129–135. 23273566 10.1016/j.tim.2012.11.005 PMC3729046

[11] Hare KM, Morris P, Smith-Vaughan H, Leach AJ (2008) Random colony selection versus colony morphology for detection of multiple pneumococcal serotypes in nasopharyngeal swabs. Pediatr Infect Dis J 27: 178–180. 18174871 10.1097/INF.0b013e31815bb6c5

[12] Huebner RE, Dagan R, Porath N, Wasas AD, Klugman KP (2000) Lack of utility of serotyping multiple colonies for detection of simultaneous nasopharyngeal carriage of different pneumococcal serotypes. Pediatr Infect Dis J 19: 1017–1020. 11055610 10.1097/00006454-200010000-00019

[13] Hansman D, Morris S, Gregory M, McDonald B (1985) Pneumococcal carriage amongst Australian aborigines in Alice Springs, Northern Territory. J Hyg (Lond) 95: 677–684. 3879259 10.1017/s0022172400060782 PMC2129567

[14] Brugger SD, Hathaway LJ, Muhlemann K (2009) Detection of Streptococcus pneumoniae strain cocolonization in the nasopharynx. J Clin Microbiol 47: 1750–1756. 19386843 10.1128/JCM.01877-08 PMC2691125

[15] Ercibengoa M, Arostegi N, Marimon JM, Alonso M, Perez-Trallero E (2012) Dynamics of pneumococcal nasopharyngeal carriage in healthy children attending a day care center in northern Spain. Influence of detection techniques on the results. BMC Infect Dis 12: 69. 22440017 10.1186/1471-2334-12-69 PMC3383471

[16] Lipsitch M, Abdullahi O, D'Amour A, Xie W, Weinberger DM, et al (2012) Estimating rates of carriage acquisition and clearance and competitive ability for pneumococcal serotypes in Kenya with a Markov transition model. Epidemiology 23: 510–519. 22441543 10.1097/EDE.0b013e31824f2f32 PMC3670084

[17] Auranen K, Mehtala J, Tanskanen A, S Kaltoft M (2010) Between-strain competition in acquisition and clearance of pneumococcal carriage–epidemiologic evidence from a longitudinal study of day-care children. Am J Epidemiol 171: 169–176. 19969530 10.1093/aje/kwp351 PMC2800239

[18] Vu HT, Yoshida LM, Suzuki M, Nguyen HA, Nguyen CD, et al (2011) Association between nasopharyngeal load of Streptococcus pneumoniae, viral coinfection, and radiologically confirmed pneumonia in Vietnamese children. Pediatr Infect Dis J 30: 11–18. 20686433 10.1097/INF.0b013e3181f111a2

[19] O'Brien KL, Nohynek H (2003) Report from a WHO working group: standard method for detecting upper respiratory carriage of Streptococcus pneumoniae. Pediatr Infect Dis J 22: 133–140. 12586977 10.1097/01.inf.0000048676.93549.d1

[20] Dhoubhadel BG, Yasunami M, Yoshida LM, Thi HA, Thi TH, et al (2014) A novel high-throughput method for molecular serotyping and serotype-specific quantification of Streptococcus pneumoniae using a nanofluidic real-time PCR system. J Med Microbiol 63: 528–539. 24464695 10.1099/jmm.0.071464-0

[21] Battig P, Hathaway LJ, Hofer S, Muhlemann K (2006) Serotype-specific invasiveness and colonization prevalence in Streptococcus pneumoniae correlate with the lag phase during in vitro growth. Microbes Infect 8: 2612–2617. 16938479 10.1016/j.micinf.2006.07.013

[22] Azzari C, Resti M (2008) Reduction of carriage and transmission of Streptococcus pneumoniae: the beneficial “side effect” of pneumococcal conjugate vaccine. Clin Infect Dis 47: 997–999. 18781870 10.1086/591967

[23] Brueggemann AB, Pai R, Crook DW, Beall B (2007) Vaccine escape recombinants emerge after pneumococcal vaccination in the United States. PLoS Pathog 3: e168. 18020702 10.1371/journal.ppat.0030168 PMC2077903

[24] Croucher NJ, Harris SR, Fraser C, Quail MA, Burton J, et al (2011) Rapid pneumococcal evolution in response to clinical interventions. Science 331: 430–434. 21273480 10.1126/science.1198545 PMC3648787

[25] Leung MH, Oriyo NM, Gillespie SH, Charalambous BM (2011) The adaptive potential during nasopharyngeal colonisation of Streptococcus pneumoniae. Infect Genet Evol 11: 1989–1995. 21925618 10.1016/j.meegid.2011.09.002

[26] Marks LR, Reddinger RM, Hakansson AP (2012) High levels of genetic recombination during nasopharyngeal carriage and biofilm formation in Streptococcus pneumoniae. MBio 3. 10.1128/mBio.00200-12 PMC3448161 23015736

[27] Lipsitch M, Dykes JK, Johnson SE, Ades EW, King J, et al (2000) Competition among Streptococcus pneumoniae for intranasal colonization in a mouse model. Vaccine 18: 2895–2901. 10812233 10.1016/s0264-410x(00)00046-3

[28] Dawid S, Roche AM, Weiser JN (2007) The blp bacteriocins of Streptococcus pneumoniae mediate intraspecies competition both in vitro and in vivo. Infect Immun 75: 443–451. 17074857 10.1128/IAI.01775-05 PMC1828380

[29] Zhang Y, Auranen K, Eichner M (2004) The influence of competition and vaccination on the coexistence of two pneumococcal serotypes. Epidemiology and Infection 132: 1073–1081. 15635964 10.1017/s0950268804002894 PMC2870198

