# Supplementary material for: Bacterial Load of Pneumococcal Serotypes Correlates with Their Prevalence and Multiple Serotypes Is Associated with Acute Respiratory Infections among Children Less Than 5 Years of Age
Source: PLoS One. 2014 Oct 31;9(10):e110777. doi: 10.1371/journal.pone.0110777 (PMC4216008; doi:10.1371/journal.pone.0110777)
Supplement: Table S2 — Data dictionary file of database of ARI and healthy children. (DOCX) [file pone.0110777.s002.docx]

README file:

This file describes the variables in the file of **data_multiple_serotype.xlsx** which was the source of results presented in the paper "Bacterial Load of Pneumococcal Serotypes Correlates with Their Prevalence and Multiple Serotypes is Associated with Acute Respiratory Infections."

If you have any queries, please contact to the corresponding author:

Dr. Lay-Myint Yoshida

E-mail: [lmyoshi@nagasaki-u.ac.jp](mailto:lmyoshi@nagasaki-u.ac.jp)

| Variable | Type | Label/description |
| --- | --- | --- |
| formid | numeric | Record identifier number |
| hosp | numeric | 1: ARI cases  0: Healthy children |
| agemonth | numeric | Age of children in months |
| agegroup | numeric | 1: <6m: age less than 6 months  2: 6-11m: age 6 to 11 months  3: 12-23m: age 12 to 23 months  4: 24-59m: age 24 to 59 months |
| gender | numeric | 1: male  0: female |
| culture_result | numeric | 1: positive: culture positive  0: culture: culture negative |
| pcr_result | numeric | 1: positive: LytA PCR positive  0: negative: LytA PCR negative |
| carriage | numeric | 1: detected: both culture and LytA PCR positive  0: undetected: in all other cases |
| lytalog10 | numeric | Total bacterial load of pneumococcus (unit: log10/microL) |
| stserotype | string | First serotype of pneumococcus (unit log10/microL) |
| stlog10 | numeric | Bacterial load of first serotype (unit log10/microL) |
| ndserotype | string | Second serotype of pneumococcus |
| ndlog10 | numeric | Bacterial load of second serotype |
| rdsero | string | Third serotype of pneumococcus |
| rdlog10 | numeric | Bacterial load of third serotype |
| multiple_sero | numeric | Detection of 2 or more serotypes;  1: detected  0: undetected |
| typeable | numeric | Serotypes typeable with the assay;  1: typeable  0: non typeable |
| prevalence | numeric | Prevalence of serotype/serogroup in ARI cases and healthy children |
| vac_sero | numeric | Serotypes present in 13 valent pneumococcal conjugate vaccine (PCV13);  1: vac_type (PCV13)  0: non_vac_type (non PCV13) |
| pcv7_sero | numeric | Serotypes present in 7 valent pneumococcal conjugate vaccine (PCV7);  1: present  0: absent |
| pcv10_sero | numeric | Serotypes present in 10 valent pneumococcal conjugate vaccine (PCV10);  1: present  0: absent |
| age_6m | numeric | Age less than 6 months (<6m) and equal or more than 6 months (>=6m);  1: <6m  2: >=6m |
| abused | numeric | Use of antibiotics before hospitalization  1: yes  2: no  3: unknown |
| daycare | numeric | Attendance of child to daycare  1: attended  0: not attended |
